# The Story of the Insane from Year to Year

**Published:** 1897-05-01

**Authors:** 


### THE STORY OF THE INSANE FROM YEAR TO YEAR.

Ninth Series.

### LINCOLN COUNTY ASYLUM.

Here the numbers were almost stationary, being 731 at the beginning of the year and 734 at the close of it. There were 170 admissions, 48 readmissions, 91 recoveries, 12 discharged relieved, and 108 deaths. Calculated on the admissions, the recoveries stand at 43·3 per cent., and the deaths work out at 14·8 on the average resident. Cerebral and spinal diseases account for 38 of the deaths, consumption for 10, and pneumonia for 13. There was one death from typhoid fever and another from dysenteric diarrhœa. Table X., which shows the causes of insanity, tells us that 118 of the year's admissions were due to hereditary tendency and 17 to drink; or, in other words, 135 of 170 should never have been in existence at all; and, although these facts are constantly being brought under the notice of the British public, the effect produced must be small indeed. Post-mortem examinations were made in 45 cases, and this number must be deemed fairly satisfactory, as Mr. Marsh is one of those conscientious medical superintendents who obtain permission from the friends before making these examinations. The asylum is full, and 63 patients are boarded out in other asylums; but a new asylum is about to be built for one of the divisions of the county. The visitors state in their report to the County Council that "their valued medical superintendent is so unwell as to render his absence on sick leave for a lengthened time absolutely necessary," and the annual report is therefore presented by the assistant medical officer, Dr. Torney. The weekly rate of maintenance is 9s. per head.

### THE MULLINGAR DISTRICT ASYLUM.

The limits of accommodation are stated to be 621, and only 617 were in the asylum, so that at first sight it would seem as if there were no overcrowding; but we find that the male side contained 53 patients too many, while there were 57 vacant beds on the female side, a condition of things which, as Dr. Finegan points out, almost never exists in English asylums. There were 114 admissions, 35 readmissions, 50 recoveries, 16 discharged relieved, and 38 deaths. The recoveries were 33·5 per cent. on the admissions, and the deaths 5·9 per cent. on the average number resident, both of these being under the average of English asylums. Brain diseases account for 6 of the deaths and consumption for 7. Moral causes, such as domestic trouble, love, and religion, are the assigned causes of the insanity in 19 of the admissions; intemperance in drink in 7, and hereditary influence in 36. It is rather curious that the mortality on the female side is nearly three times as high as on the male side, and yet Dr. Finegan tells us that the supervision, nursing, and general order are superior on the female side. The asylum has been heated on the plenum system, and it is anticipated that the death-rate from consumption will be reduced. This we doubt, and if Dr. Finegan will read Dr. Jones' account of the system as carried out at Claybury he will probably doubt it also; but it will take many years to prove it, and much depends on whether the system is solely relied on or whether open fireplaces are still kept in use. We are pleased to learn that a lady medical has been appointed as assistant medical officer, and that this appointment has been entirely successful. The rate of maintenance is £26 12s. 9d. per annum.

### GLOUCESTER COUNTY ASYLUMS.

The numbers fell during the year from 1,069 to 1,059. There were 261 admissions; 54 readmissions; 127 recoveries; 38 discharged relieved, and 123 deaths. The recoveries stand at 44·87 on the admissions, and the deaths at 11·71 on the average number resident. Both of these percentages are above the average. Of the deaths 55 were caused by cerebral and spinal disease; 8 by consumption; and 13 by pneumonia. The last-named disease being a factor in 7 other deaths. Hereditary influence contributed to the insanity in 16 of the admissions only, and intemperance scarcely figures at all; but, as the causes are only given in 84 out of 261 admissions, the table has no practical value. Mr. Craddock points out that although the numbers had decreased during the year, the decrease was owing to the transference of out-county patients to another asylum, and that there was really an increase of 13 Gloucestershire patients. Mr. Craddock deplores this increase in the number of patients, which is going on all over England; and he hits the right nail on the head when he says that the evil will increase "so long as no steps are taken to check the liberty which the mentally, morally, and physically tainted possess, along with the best and strongest, of adding to the numbers of the population replicas of themselves." Indeed, it is quite time that this serious question was taken up by our legislators. In the meantime we hope Mr. Craddock's words will find a wide circulation. The visitors say that "under the able management of Dr. Craddock, the asylums have been maintained in good order." The average weekly cost per head was 7s. 5⅝d.

